# The impact of pulse oximetry on diagnosis, management and outcomes of acute febrile illness in low-income and middle-income countries: a systematic review

**DOI:** 10.1136/bmjgh-2021-007282

**Published:** 2021-11-25

**Authors:** Rusheng Chew, Meiwen Zhang, Arjun Chandna, Yoel Lubell

**Affiliations:** 1 Economics and Implementation Research Group, Mahidol Oxford Tropical Medicine Research Unit, Bangkok, Thailand; 2 Centre for Tropical Medicine and Global Health, University of Oxford, Oxford, UK; 3 Angkor Hospital for Children, Cambodia Oxford Medical Research Unit, Siem Reap, Cambodia

**Keywords:** other diagnostic or tool, health services research, public health, infections, diseases, disorders, injuries, systematic review

## Abstract

**Background:**

Acute fever is a common presenting symptom in low/middle-income countries (LMICs) and is strongly associated with sepsis. Hypoxaemia predicts disease severity in such patients but is poorly detected by clinical examination. Therefore, including pulse oximetry in the assessment of acutely febrile patients may improve clinical outcomes in LMIC settings.

**Methods:**

We systematically reviewed studies of any design comparing one group where pulse oximetry was used and at least one group where it was not. The target population was patients of any age presenting with acute febrile illness or associated syndromes in LMICs. Studies were obtained from searching PubMed, EMBASE, CABI Global Health, Global Index Medicus, CINAHL, Cochrane CENTRAL, Web of Science and DARE. Further studies were identified through searches of non-governmental organisation websites, snowballing and input from a Technical Advisory Panel. Outcomes of interest were diagnosis, management and patient outcomes. Study quality was assessed using the Cochrane Risk of Bias 2 tool for Cluster Randomised Trials and Risk of Bias in Non-randomized Studies of Interventions tools, as appropriate.

**Results:**

Ten of 4898 studies were eligible for inclusion. Their small number and heterogeneity prevented formal meta-analysis. All studies were in children, eight only recruited patients with pneumonia, and nine were conducted in Africa or Australasia. Six were at serious risk of bias. There was moderately strong evidence for the utility of pulse oximetry in diagnosing pneumonia and identifying severe disease requiring hospital referral. Pulse oximetry used as part of a quality-assured facility-wide package of interventions may reduce pneumonia mortality, but studies assessing this endpoint were at serious risk of bias.

**Conclusions:**

Very few studies addressed this important question. In LMICs, pulse oximetry may assist clinicians in diagnosing and managing paediatric pneumonia, but for the greatest impact on patient outcomes should be implemented as part of a health systems approach. The evidence for these conclusions is not widely generalisable and is of poor quality.

**Key questions**

**What is already known?**

- Acute fever is a common presenting symptom in low/middle-income countries (LMICs); both fever and hypoxaemia are associated with sepsis, which may not necessarily be from a respiratory infection.
- Pulse oximetry may, thus, be clinically useful in the assessment and management of acutely febrile patients in LMICs.
- Several studies in LMICs have examined the impact of pulse oximetry on clinical outcomes in various infective syndromes, but no synthesis of their findings has been attempted.

**What are the new findings?**

- There is moderately strong evidence that pulse oximetry is beneficial in identifying severely ill paediatric patients who would benefit from escalation of care, but scant high-quality evidence for its benefit in terms of patient outcomes, in particular mortality.
- There are major gaps in the evidence base, with a limited range of LMIC contexts studied and the bulk of the available evidence coming from biased non-randomised studies.

**What do the new findings imply?**

- To obtain maximum benefit from pulse oximetry in assessing and managing acutely febrile patients in LMICs, pulse oximetry must be introduced as part of a quality-assured system-wide approach.
- Policymakers must consider the specific context in which pulse oximetry will be used and tailor the intervention accordingly.
- Future research must address the utility of pulse oximetry in patient populations under-represented or not represented in our review, especially adults, patients living at high altitudes, patients with darker skin and patients living in Asian LMICs.

## Introduction

Acute fever is one of the most common reasons for seeking medical attention in low/middle-income countries (LMICs).^1^ It is highly associated with sepsis, a medical emergency characterised by dysregulation of the host inflammatory response to infection.^2^ It is, thus, imperative that patients presenting to health facilities with acute febrile illness at high risk of sepsis are identified early to enable escalation to higher level care and improve clinical outcomes. Hypoxaemia has been shown to be highly prevalent in such patients and a predictor of disease severity,^3–6^ but clinical examination is an insensitive tool for its detection.^7^ As such, the use of pulse oximetry in all age groups to detect abnormally low peripheral oxygen saturation (SpO_2_) is now well-established in high-income countries (HICs), leading to it being dubbed the ‘fifth vital sign’.^8 9^

Fever is a non-specific sign present in both severe and mild illness, and hypoxaemia is increasingly recognised as a clinical feature of sepsis also from non-respiratory causes such as malaria.^10–12^ It is common for patients to have overlapping clinical signs and symptoms of acute respiratory and non-respiratory infections concurrently.^13^ Therefore, incorporation of peripheral oxygen saturation measurement into guidelines based principally on clinical examination, such as the WHO’s Integrated Management of Childhood Illness (WHO IMCI) algorithms, may aid healthcare workers in low-resource settings identify severely ill patients and formulate management plans with a greater degree of confidence and accuracy.

Pulse oximetry is not widely used in LMICs, especially in rural areas where most people live and, hence, where it is likely to be most useful.^14^ Efforts are underway to increase access to pulse oximetry in LMICs, aided partly by the availability of durable low-cost oximeters and galvanised by the WHO recommendation to use them in hospitals and primary health centres,^15^ with the COVID-19 pandemic providing extra impetus. However, there is little high-quality evidence of their utility in these settings.^16^ The contexts in which health services are provided in LMICs differ considerably from those in HICs, for instance in terms of staff-to-patient ratios, staff levels of training and health facility infrastructure. Therefore, the benefits of pulse oximetry, especially in refining diagnosis and treatment,^8 9^ seen in HICs may not be fully generalisable to LMICs, given the absence of some or all the necessary mediators, such as reliable supplemental oxygen and trained staff, in the latter. In the better-resourced health systems of HICs the potential benefits of an intervention will often be more fully realised than in LMICs where suboptimal contextual aspects might diminish the effectiveness or even feasibility of pulse oximetry.

In this systematic review, we aimed to assess the impact of pulse oximetry on diagnosis, clinical management and outcomes of patients with acute febrile illness in LMICs. The review was motivated by our multi-faceted programme on acute febrile illness in rural South and Southeast Asia,^14^ and specifically by our interest in implementation studies on the use of pulse oximeters in these settings.

## Methods

### Protocol and registration

This systematic review was performed according to a prespecified protocol, which was registered on the PROSPERO database (registration number CRD42021257241).^17^ The report was prepared in accordance with Preferred Reporting Items for Systematic Reviews and Meta-Analyses (PRISMA) 2020 guidelines,^18^ and the completed PRISMA 2020 checklist can be found in online supplemental appendix S1.

10.1136/bmjgh-2021-007282.supp1 Supplementary data

### Eligibility criteria

Observational and interventional studies conducted in LMICs designed with one group where pulse oximetry was used and at least one comparator group where pulse oximetry was not used were eligible for inclusion. The target population was adults, children and neonates presenting with acute febrile illness, or syndromes associated with acute febrile illness such as pneumonia or malaria, to a healthcare worker or facility of any type or level. Modelling studies, studies in which the participants were patients undergoing perioperative care, or studies in which pulse oximetry was used for asymptomatic screening were excluded. The outcomes of interest were diagnosis, management (including, but not limited to, further investigation, treatment and onward referral) and patient outcomes (including, but not limited to, morbidity and mortality) of acute febrile illness or an associated clinical syndrome.

### Search strategy

We searched the following databases on 29 June 2021 with no restriction on language or date of publication: PubMed, EMBASE, CABI Global Health, Global Index Medicus, CINAHL, Cochrane CENTRAL and Web of Science. The search strings used for each of these databases are available in online supplemental appendix S2. We also searched the websites of the WHO, the International Committee of the Red Cross and Médecins Sans Frontières to identify relevant grey literature.

10.1136/bmjgh-2021-007282.supp2 Supplementary data

To identify further studies for inclusion, we searched reference lists of any relevant systematic reviews returned from the above searches, as well as a search of the Database of Abstracts of Reviews of Effects. In addition, we applied backward and forward snowballing (using the Google Scholar citation tracking facility) to included studies and searched the publication lists of first and last authors of included studies as per Google Scholar. We assembled a Technical Advisory Panel who peer-reviewed this search strategy. Advisors were also asked to identify obvious omissions in the list of included articles, suggest additional authors whose publication lists were examined and suggest key practitioners who were approached for further studies, including grey literature (online supplemental appendix S3).

10.1136/bmjgh-2021-007282.supp3 Supplementary data

### Study selection

Two reviewers (RC and MZ) independently screened the titles and abstracts of the studies returned by database, website, snowball and hand searches, as well as recommendations arising from Technical Advisory Panel input, applying the aforementioned inclusion and exclusion criteria. If there was ambiguity about whether a study should be included from the title and abstract alone, the full text of the study was obtained for further assessment. Full texts of all studies identified through title and abstract screening were read to produce a final list of included studies and reasons for rejection recorded. The list of studies rejected at this stage and reasons for rejection can be found in online supplemental appendix S4. Disagreement between reviewers was resolved by discussion in the first instance. A third reviewer (AC), whose decision was final, was asked to adjudicate if required.

10.1136/bmjgh-2021-007282.supp4 Supplementary data

### Data extraction

One reviewer (RC) independently extracted data from each study into a Microsoft Excel spreadsheet based on the fields in the Cochrane data collection form for intervention reviews for randomised controlled trials (RCTs) and non-RCTs.^19^ A second reviewer (AC) checked the accuracy of the extracted data for each included study. Any discrepancies were resolved by discussion. The data extraction template can be found in online supplemental appendix S5.

10.1136/bmjgh-2021-007282.supp5 Supplementary data

### Data analysis and synthesis of results

Meta-analysis of data to produce pooled ORs and risk ratios was precluded by the small number and heterogeneity of included studies, as well as the paucity of randomised studies.^20^ We, therefore, tabulated relevant data from each study and performed a narrative synthesis to draw conclusions, taking into consideration differences in study design and setting.

### Quality assessment

We used the Cochrane Risk of Bias 2 tool for Cluster Randomised Trials (RoB 2 CRT) to assess risk of bias for cluster-randomised trials,^21^ and the Cochrane Risk of Bias in Non-randomized Studies of Interventions (ROBINS-I) tool to assess risk of bias for non-randomised studies.^22^ Each study was assessed by two independent reviewers (RC and one of either MZ or AC), with disagreements resolved through discussion in the first instance. A third reviewer (either MZ or AC), whose decision was final, was asked to adjudicate if required. Risk of bias for cluster-randomised trials was categorised as either ‘low’, ‘some concerns’ or ‘high’ as per RoB 2 CRT, while risk of bias for non-randomised studies was categorised as either ‘low’, ‘moderate’, ‘serious’ or ‘critical’ as per ROBINS-I.

### Role of funding sources

The funders had no role in study design, data collection, data analysis, data interpretation or writing of the manuscript. All authors had full access to the data and had final responsibility for the decision to submit the manuscript for publication.

### Patient and public involvement

No patients or members of the public were directly involved in the conduct of this work.

## Results

**Figure 1:**
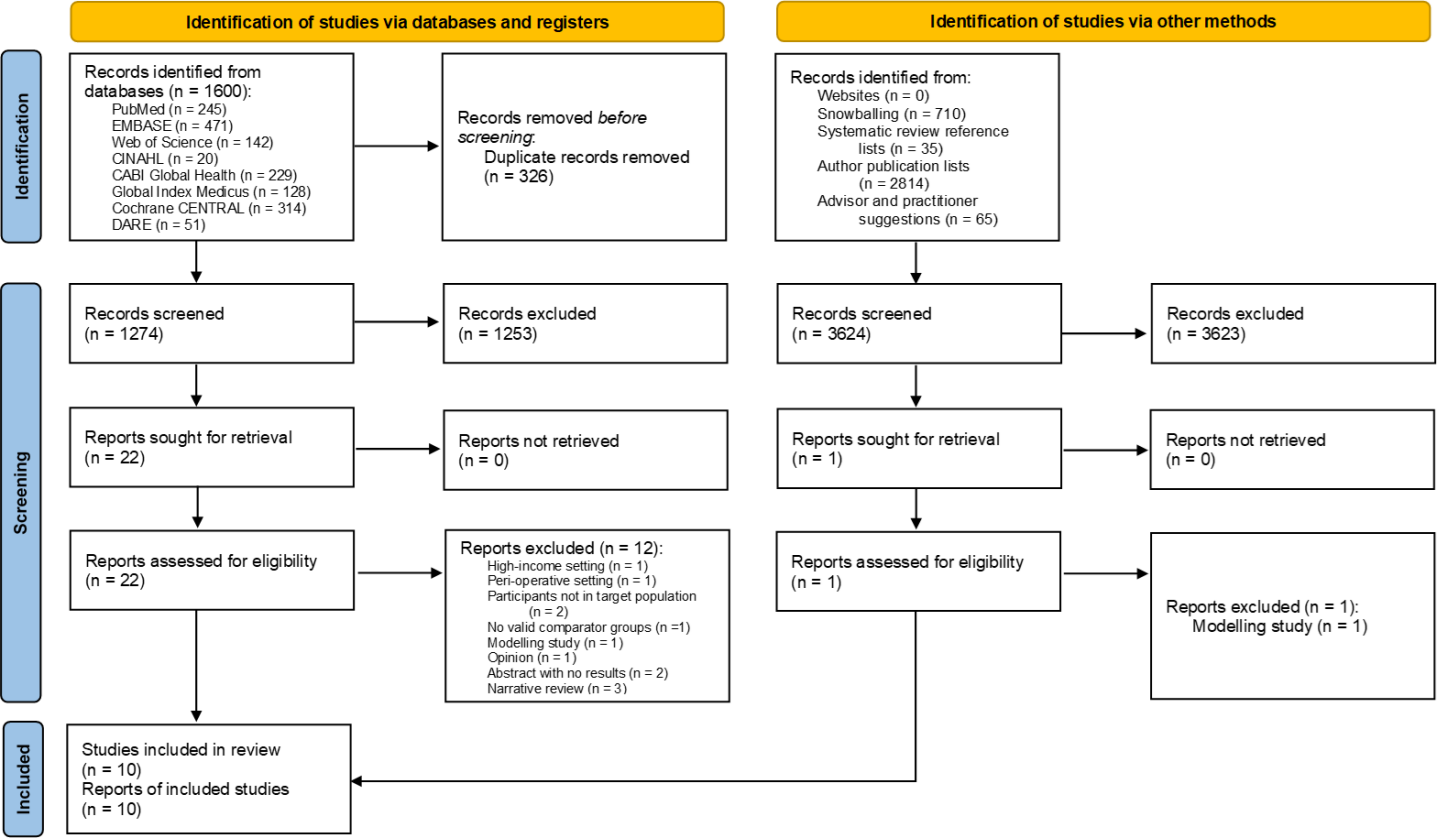
Review Preferred Reporting Items for Systematic Reviews and Meta-Analyses 2020 flow diagram.

A total of 4898 studies were considered for inclusion. Ten were ultimately selected, as per the PRISMA flow diagram in figure 1.

All studies enrolled children aged <15 years. Six studies included, but not exclusively, patients <2 months old^23–28^; another enrolled only those in this age group.^29^ Seven studies included patients aged 2 months to ≤5 years,^23–28 30–32^ and only two included children >5 years and adolescents.^26 28^ Only two studies were randomised trials,^26 31^ and, for the purposes of this review, only two evaluated the use of pulse oximetry in non-respiratory infections^23 26^; the clinical syndrome of interest in the remainder was community-acquired pneumonia or acute lower respiratory tract infection.^24–32^ In three studies pulse oximetry was used as part of a set of interventions, all of which included varying improvements to oxygen provision.^25 26 28^

Three studies were conducted in Papua New Guinea,^24 25 28^ one in Peru^30^ and six in sub-Saharan Africa.^23 27 29 31 32^ Five studies were conducted in rural primary care settings,^23 27 29 31 32^ three in rural hospitals^24 25 28^ and two in urban hospitals.^26 30^ All studies used p<0.05 as the threshold for statistical significance, except for Madico *et al* where statistical significance was not assessed.^30^

### Quality of included studies

**Figure 2:**
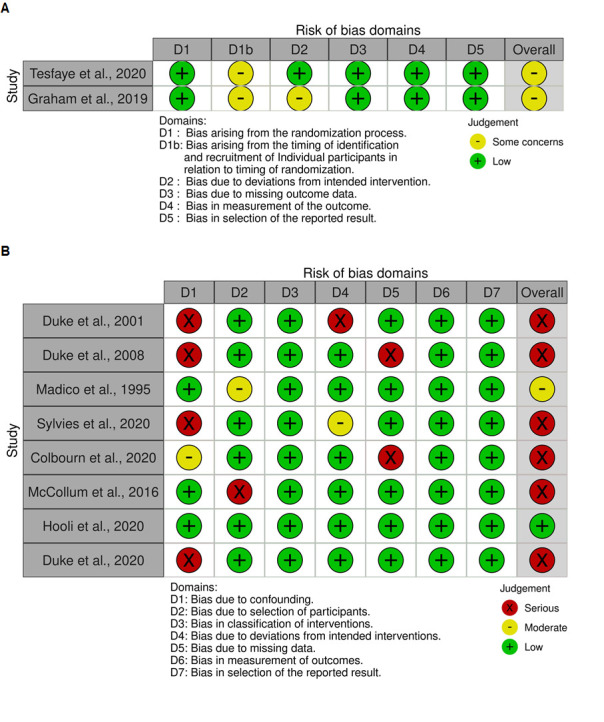
Risk of bias assessments of (A) included cluster-randomised trials, performed using the Cochrane Risk of Bias 2 tool for Cluster-Randomised Trials and (B) included non-randomised studies, performed using the Cochrane Risk of Bias in Non-randomized Studies of Interventions tool.

The risk of bias assessment for each study is shown in figure 2A (for cluster-randomised trials) and figure 2B (for non-randomised studies), which were produced using the *robvis* tool.^33^ With four exceptions,^26 29–31^ all studies were at serious risk of bias. Four of the eight non-randomised studies were at serious risk of bias due to confounding solely or in combination with other domains,^23–25 27^ while one was at serious risk of bias due to missing data,^27^ and another due to selection bias.^32^ The risk of bias of both cluster-randomised trials was moderate.^26 31^

Notably, two studies did not supply appropriately sized probes and reported considerable staff difficulty using pulse oximeters.^29 32^ This contributed to inaccurate or non-recording of SpO_2_.

### Impact of pulse oximetry on diagnosis

Two studies assessed the diagnostic impact of pulse oximetry, the principal findings of which are described below and their characteristics in table 1. The strength of the evidence presented in this section is moderate.

**Table 1:** Impact of pulse oximetry on diagnosis of acute febrile illness or associated syndromes

Study	Study design	Syndrome	Intervention and comparison groups	Outcome measure	Participants	Age range	Setting	Country (continent)	Reported effect
Madico *et al*^30^	Prospective cohort study	ALRI	PO vs WHO algorithm^34^ vs PO+WHO algorithm vs clinical diagnosis*	Independent third-party clinical diagnosis of ALRI made without pulse oximetry	269 children with ARI	2–60 months	Single urban hospital paediatric emergency department	Peru (South America)	125/160 (78.1%) diagnosed in PO group vs 130/160 (81.3%) in WHO algorithm group vs 150/160 (93.8%) in PO+WHO algorithm group
Tesfaye *et al*^31^	Parallel cluster-randomised trial	Pneumonia	PO+IMCI vs IMCI†^35^	Clinical diagnosis of severe pneumonia	1804 children with cough or difficulty breathing <14 days	2–59 months	24 rural primary health centres	Ethiopia (Africa)	148/928 (15.9%; 95% CI 4.7% to 27.2%) diagnosed in PO+IMCI group vs 34/876 (3.9%; 95% CI 1.2% to 6.6%) in IMCI group, p<0.001 Adjusted OR 5.4 (95% CI 2.0 to 14.3), p=0.001

Hypoxaemia was defined as peripheral oxygen saturation (SpO_2_) <90% unless indicated otherwise.

*Hypoxaemia defined as SpO_2_<96.6%.

†Intervention group included a staff training component on the use of pulse oximetry.

ALRI, acute lower respiratory tract infection (pneumonic and non-pneumonic); ARI, acute respiratory infection; IMCI, WHO Integrated Management of Childhood Illness guideline 2014; PO, pulse oximetry.

In a small single-hospital prospective cohort study, Madico *et al* found that the yield of pulse oximetry alone for the diagnosis of acute lower respiratory tract infection in children aged 2–60 months presenting to a Peruvian urban paediatric emergency department was comparable with that of the 1990 WHO diagnostic guideline at approximately 80%,^34^ using third-party independent clinical diagnosis as a proxy reference standard. The third-party independent clinical diagnosis was made without pulse oximetry. When both pulse oximetry and guideline were used together, near-perfect (94%) concordance with clinical diagnosis was achieved.^30^

The diagnostic utility of pulse oximetry in a rural primary care setting was also demonstrated by Tesfaye *et al*, who conducted a cluster-randomised trial in 24 primary health centres in Malawi. There was a significant fivefold increase in the adjusted odds of diagnosing severe pneumonia in similarly aged symptomatic children when pulse oximetry with cut-off SpO_2_ <90% was used in conjunction with the 2014 WHO IMCI guideline, compared with guideline alone.^31^

### Impact of pulse oximetry on clinical management

Four studies assessed the impact of pulse oximetry on clinical management (table 2). The strength of the evidence presented in this section is low-moderate.

**Table 2:** Impact of pulse oximetry on management of acute febrile illness or associated syndromes

Study	Study design	Syndrome	Intervention and comparison groups	Outcome measure	Participants	Age range	Setting	Country (continent)	Reported effect
McCollum *et al*^32^	Retrospective secondary analysis of prospective cohort study data	Pneumonia	PO+IMCI vs PO+Malawi guideline 2000 vs IMCI^35^ vs Malawi guideline 2000^36^*	Missed eligibility for referral to hospital	14092 children with pneumonia	2–59 months	18 rural health centres and 38 village clinics	Malawi (Africa)	At rural health centres, IMCI resulted in 390/938 (41.5%) missed eligible referrals and Malawi guideline 2000 in 143/1761 (8.1%) if PO not used At village clinics, both IMCI and Malawi guideline 2000 resulted in 52/990 (5.3%) missed eligible referrals if PO not used
Hooli *et al*^29^	Retrospective secondary analysis of prospective cohort study data	Pneumonia	PO+IMCI vs PO+Malawi guideline 2000 vs IMCI^35^ vs Malawi guideline 2000^36^*	Missed eligibility for referral to hospital	261 infants with cough and/or shortness of breath	<2 months	18 rural health centres	Malawi (Africa)	IMCI resulted in 1/238 (0.4%) missed referral if PO not used, and Malawi guideline 2000 1/236 (0.4%) If hypoxaemia was defined as SpO_2_ <93%, IMCI resulted in 2/238 (0.8%) missed referral if PO not used, and Malawi guideline 2000 4/236 (1.7%)
Tesfaye *et al*^31^	Parallel cluster-randomised trial	Pneumonia	PO+IMCI vs IMCI^35^*	Referral to hospital	1804 children with cough or difficulty breathing <14 days	2–59 months	24 rural primary health centres	Ethiopia (Africa)	116/148 (78.4%, 95% CI 67.6 to 89.2) referred in PO+IMCI group vs 15/34 (44.1%, 95% CI 6.9 to 81.3) in IMCI group, p=0.496
Sylvies *et al*^23^	Uncontrolled before-and-after study	Non-malarial fever	PO+IMCI vs IMCI^35^ vs Malawi guideline 2019 vs Malawi guideline 2016*†	Antibiotic prescription	3504 children with non-malarial fever	0–59 months	Five mobile rural health clinics	Malawi (Africa)	336/795 (42.2%) prescribed antibiotics in PO+IMCI group vs 150/178 (84.2%) in IMCI group vs 485/571 (84.9%) in Malawi guideline 2019 group vs 1461/1960 (74.5%) in Malawi guideline 2016 group, p<0.001 OR for reduced antibiotic prescription in PO+IMCI group vs IMCI group 7.3 (95% CI 4.8 to 11.4); vs Malawi guideline 2019 group 7.9 (95% CI 6.1 to 10.5); vs Malawi guideline 2016 group 4.0 (95% CI 3.3 to 4.7)

All interventions incorporating pulse oximetry included a staff training component. Hypoxaemia was defined as peripheral oxygen saturation (SpO_2_) <90% unless indicated otherwise.

*Intervention groups with pulse oximetry included a staff training component on the use of pulse oximetry.

†Hypoxaemia defined as SpO_2_ <95%.

IMCI, WHO Integrated Management of Childhood Illness guideline 2014; PO, pulse oximetry.

Three of these studies examined the effect of pulse oximetry in addition to various contemporaneous clinical guidelines on referral to hospital from primary care for severe pneumonia. All were conducted in sub-Saharan Africa and demonstrated increased referral rates in older children when pulse oximetry was used.^29 31 32^ However, compliance with referral decisions was inconsistent.^31 32^

In keeping with their finding of significantly increased odds of diagnosing severe pneumonia with the combined use of pulse oximetry and 2014 IMCI guideline, Tesfaye *et al* also reported that the proportion of children referred to the hospital for severe pneumonia in the intervention group was nearly two times that in the comparator group. Importantly, only approximately half the children referred in the intervention group and three-quarters of those in the comparator group actually presented to the hospital.^31^

In a comparable rural Malawian primary care setting, McCollum *et al* also showed that pulse oximetry had the potential to reduce missed referrals to the hospital in a large cohort of similarly aged children with pneumonia. Between 5% and 42% of children with severe disease would not have been deemed eligible for referral in the absence of pulse oximetry, depending on the sensitivity of the guideline used and whether the children were seen at health centres or mobile village clinics. The SpO_2_ cut-off was <90%; had the cut-off been <93% to include children with moderate hypoxaemia, the proportion of missed eligible referrals would have been in the range of 20%–62%. However, like in the study by Tesfaye *et al*, even with pulse oximetry only 48% of patients eligible for referral were actually referred, indicating that factors other than clinical assessment play a role in clinician and caregiver decision-making.^32^

Hooli *et al* applied the same methodology and SpO_2_ cut-off of <90% as McCollum *et al* to 261 neonates and infants <2 months in the same Malawian settings, and found that both national and IMCI guidelines missed less than 1% of cases who should have been referred in the absence of pulse oximetry.^35 36^ When the cut-off was increased to <93%, the national Malawian guideline missed just under 2% while the WHO IMCI guideline missed approximately 1%.^29^

Finally, Sylvies *et al* conducted an uncontrolled before-and-after study comparing antibiotic prescription rates for non-malarial febrile illness in children <5 years in five Malawian mobile rural health clinics. Pulse oximetry used together with the IMCI guideline was compared with three comparators: IMCI guideline, 2016 national guideline and 2019 national guideline. Antibiotic prescription rates were reduced by approximately half in the pulse oximetry plus IMCI group compared with the others. When compared with IMCI alone, the addition of pulse oximetry increased the odds of reducing antibiotic prescription over sevenfold, despite only 30% of children in the intervention group having had SpO_2_ measured.^23^

### Impact of pulse oximetry on patient outcomes

Six studies assessed the impact of pulse oximetry on patient outcomes (table 3). The strength of the evidence presented in this section is low.

**Table 3:** Impact of pulse oximetry on outcomes of acute febrile illness or associated syndromes

Study	Study design	Syndrome	Intervention and comparison groups	Outcome measure	Participants	Age range	Setting	Country (continent)	Reported effect
Duke *et al*^24^	Uncontrolled before-and-after study	Community-acquired pneumonia	PO vs clinical signs*	30-day mortality	961 children with severe or very severe pneumonia	>28 days and <5 years	Single rural hospital at high altitude	Papua New Guinea (Australasia)	Mortality rate in PO group 46/703 (6.5%) vs 26/258 (10%) in clinical signs group RR 0.65 (95% CI 0.41 to 1.02, p=0.07)
Duke *et al*^25^	Uncontrolled before-and-after study	Community-acquired pneumonia	PO+improved oxygen system vs standard care†	In-hospital mortality	11291 children with pneumonia	>1 month and <5 years	Five rural hospitals	Papua New Guinea (Australasia)	Mortality in intervention group 133/4130 (3.22%, 95% CI 2.7% to 3.8%) vs 356/7161 (4.97%, 95% CI 4.5 to 5.5) in comparison group RR 0.65 (95% CI 0.52 to 0.78), p<0.0001
Graham *et al*^26^	Stepped-wedge cluster RCT	ALRI, malaria, AFE, neonatal sepsis	PO+improved oxygen system vs PO+standard care vs standard care§	Mortality (in-hospital or discharged expected to die)	3828 children with ALRI, 6113 children with AFE, 11 092 children with malaria and 7515 neonates with sepsis‡	0 days to <15 years	Nine general and three paediatric/maternity urban hospitals	Nigeria (Africa)	ALRI: aOR for PO+standard care vs standard care 0.33 (95% CI 0.12 to 0.92), p=0.035; for PO+improved oxygen package vs PO+standard care 1.42 (95% CI 0.60 to 3.36), p=0.427 Malaria, AFE and neonatal sepsis: neither PO+improved oxygen system nor PO+standard care significantly affected aORs for mortality from these conditions vs standard care
Colbourn *et al*^27^	Prospective data linkage study	Community-acquired pneumonia	PO+Malawi guideline 2000 vs Malawi guideline 2000†^36^	30-day mortality	13814 children with pneumonia	0–59 months	Mobile rural village clinics run by 38 community health workers and 18 rural health centres	Malawi (Africa)	PO would have identified 1/16 (6%) additional death at mobile rural village clinic level compared with Malawi guideline 2000 regardless of whether hypoxaemia was defined as SpO_2_ <90% or <93% PO would not have identified any additional deaths at rural health centre level compared with Malawi guideline 2000 regardless of whether hypoxaemia was defined as SpO_2_ <90% or <93%
Duke *et al*^28^	Uncontrolled before-and-after study	Community-acquired pneumonia	PO+improved oxygen system+QI vs Standard care†	In-hospital mortality	18933 neonates and children with pneumonia‡	0–13 years	36 rural hospitals	Papua New Guinea (Australasia)	IR for deaths per 100 pneumonia admissions in intervention group 1.17 (95% CI 0.48 to 1.86) vs 2.83 (95% CI 1.98 to 4.06) IRR 0.41 (95% CI 0.24 to 0.71, p<0.005)
Tesfaye *et al*^31^	Parallel cluster-randomised trial	Community-acquired pneumonia	PO+IMCI vs IMCI†^35^	Treatment failure at 14 days§	1804 children with cough or difficulty breathing <14 days	2–59 months	24 rural primary health centres	Ethiopia (Africa)	132/928 (14.2%, 95% CI 6.0 to 22.4) failed treatment in the PO+IMCI group vs 93/876 (10.6%, 95% CI 5.2 to 16.1) in the IMCI group, p=0.622

Hypoxaemia was defined as peripheral oxygen saturation (SpO_2_) <90% unless indicated otherwise.

*Hypoxaemia defined as SpO_2_ <85%.

†Intervention groups with pulse oximetry included a staff training component.

‡Prespecified subgroup analysis.

§Defined as the development or persistence of general danger signs, persistence of fever, persistence of tachypnoea, chest wall indrawing, presence of persistent cough, recurrence of fever, withdrawal from the trial or death.

AFE, acute febrile encephalopathy; ALRI, acute lower respiratory infection; aOR, adjusted OR; IMCI, WHO Integrated Management of Childhood Illness 2014; IR, incidence rate; IRR, incidence rate ratio; PO, pulse oximetry; QI, quality improvement; RCT, randomised controlled trial; RR, risk ratio.

Five studies used mortality as their outcome measure, which was measured at different time points in the patient journey. Pulse oximetry was trialled as a component of intervention sets of varying sophistication across the different study settings.^24–28^ Effect sizes ranged from negligible to considerable, and appeared to be largely influenced by study context and setting as well as the level of system-wide support included in the intervention.

Duke and collaborators performed a series of uncontrolled before-and-after studies in various rural Papua New Guinean hospital settings, commencing with a study in a highland hospital comparing the use of pulse oximetry versus clinical signs to guide supplemental oxygen administration in children <5 years with severe or very severe pneumonia. Hypoxaemia was defined as SpO_2_ <85% due to the altitude of the hospital. Thirty-day mortality was reduced by 35% following the use of pulse oximetry, but this was not statistically significant.^24^ Building on this finding, they conducted a much larger study involving similarly aged children admitted with pneumonia of any severity to five dissimilar hospitals. Overall, pulse oximetry as a component of an improved oxygen system significantly reduced the in-hospital mortality rate by over a third compared with standard care, but mortality rate reductions were seen in only two out of the five hospitals.^25^ Their most recent study, conducted across 36 hospitals, also showed a significant 59% reduction in in-hospital pneumonia mortality in neonates and older children, with pulse oximetry being introduced as part of an oxygen system supported by continuous electricity supply and quality improvement processes compared with standard care. Again, this improved outcome was seen in most, but not all, hospitals, with the variability related to facility baseline rates of pneumonia admissions, baseline quality of clinical care and sustainability of the intervention.^28^ It is also important to note that the mortality reductions associated with the introduction of pulse oximetry were realised in the context of substantial improvements to the hard and soft infrastructure of the health facilities.

A stepped-wedge cluster randomised trial by Graham *et al* in 12 urban Nigerian hospitals with a two-stage intervention comprising first of pulse oximetry, followed by its incorporation into an improved oxygen system similar to the one in the study by Duke *et al*,^28^ was helpful in addressing the question of the contribution of pulse oximetry alone in reducing child and neonatal mortality. Augmentation of standard care with pulse oximetry significantly reduced in-hospital mortality from paediatric acute lower respiratory tract infection by 67% in a prespecified subgroup analysis of 3828 children aged between 28 days and 14 years. However, the improved oxygen system had no significant additional impact on this outcome. Neither pulse oximetry nor the improved oxygen system had any significant impact on the severity-adjusted odds of mortality from paediatric malaria, paediatric acute febrile encephalopathy and neonatal sepsis.^26^

Colbourn *et al* undertook a prospective data linkage study assessing the potential impact of pulse oximetry on mortality from community-acquired pneumonia in children <5 years in a rural Malawian primary care setting. Supplementation of clinical sign-based guidelines with pulse oximetry would have identified an additional death (1/16, 6%) from mobile rural village clinics missed by guidelines alone but no additional deaths from rural health centres, further indicating a possible role for pulse oximetry in identifying children at high risk of deterioration in the community. Hypoxaemia was defined as SpO_2_ <90%, but increasing the threshold to <93% did not identify any additional deaths at either mobile village clinic or rural health centre level. However, these results should be interpreted with caution as only 6% of mobile village clinic episodes and 11% of rural health centre episodes were linked to mortality data.^27^

Finally, Tesfaye *et al* examined the effect of pulse oximetry on treatment failure of severe pneumonia at 14 days, finding no significant difference between the intervention and comparator groups. The authors state that this finding may be due to the substantial proportion of children in both groups who were referred to the hospital but did not go, primarily due to lack of transport.^31^

## Discussion

This systematic review highlights the potential benefits of pulse oximetry in the assessment of acute febrile illness by healthcare staff of various skill levels working in diverse LMIC primary and secondary care settings, but also the paucity of high-quality studies addressing diagnostic, clinical management and patient outcome endpoints. We found moderately strong evidence for the use of pulse oximetry to aid the diagnosis of pneumonia in children and referral to hospital of those with severe respiratory illnesses.^30 31^ In contrast, there was scant evidence of its benefit in other syndromes associated with acute fever, with only one study at serious risk of bias reporting a reduction in empirical antibiotic prescription.^23 26^ Several studies, mainly in hospital settings, reported a mortality benefit, but all save one were compromised by confounding (in particular secular trends) or other biases,^26^ indicating a disconnect between the certainty of evidence for a diagnostic and prognostic benefit of pulse oximetry. The generalisability of the included results beyond paediatric patients is poor, because no studies recruited adults and although six studies enrolled patients <2 months, only one focused exclusively on neonates and very young infants.^29^ Moreover, no studies were conducted in Asia where the burden of infectious diseases remains disproportionately high,^37^ and where most recent emerging respiratory infections of public health importance have originated.^38 39^ It is critical to note that the bulk of studies reporting beneficial effects of pulse oximetry used it as part of a bundle of linked interventions, underscoring the importance of a systems approach.

Notwithstanding the weak nature of the evidence, we have drawn some conclusions relevant to clinical practice and health policy. First, given its likely value in identifying the severely ill febrile child,^31^ the protocol-driven use of pulse oximetry in secondary care settings where oxygen is available may reduce morbidity and mortality in this cohort through targeting of oxygen and nursing resources.^24–26 28^ However, any such resource re-allocation must not detract from the care of less ill patients whose capacity to benefit may be adversely affected, thus negating any potential overall benefit. Second, while pulse oximetry may assist primary and acute care clinicians in identifying and managing severe disease, uncovering the considerable hidden burden of severe pneumonia has implications for health service provision and hospital resourcing to avoid overstretching secondary healthcare facilities.^31^ This is particularly relevant in settings where acute fever is commonly attributed to other causes, such as malaria.^40^ The current evidence shows that this is countered by the low uptake of hospital referral decisions, which is common in resource-limited settings but predisposes to excess morbidity and mortality. Therefore, pulse oximetry must be supplemented by efforts to increase the uptake of hospital referral recommendations or, alternatively, to increase the availability of oxygen, parenteral antibiotics and close observation in rural health centres. Barriers to accessing higher-level care—not just infrastructural but also psychosocial, such as competing priorities and perceived staff incompetence—may erode the benefits of pulse oximetry in terms of patient outcomes, as was demonstrated by Tesfaye *et al*.^31^ The observations relevant to these two conclusions strongly underline the importance of taking a system-wide view if pulse oximetry is to be implemented, especially in health systems which may not be robust. Third, customising pulse oximetry to the setting in which it is to be used is of utmost importance, since contextual factors directly impact on feasibility. For example, pulse oximetry will play different roles in primary versus higher level health facilities; in the former, it may aid gatekeeping by identifying those who can be managed in the community. However, in primary care settings the prevalence of hypoxaemia will be low, necessitating measures to minimise the risk of false positives. The resource limitations present in LMICs inevitably add a further layer of complexity to these considerations. Regardless, the utility of pulse oximetry is likely to be optimal in settings with a rigorous quality assurance programme and that actively invest in staff upskilling to ensure the sustainability of the intervention, especially in settings with low staff numbers per facility.^25 26 28^ Staff should be given appropriate and ongoing training not just in the mechanics of pulse oximetry but also in its use in syndromic diagnosis and clinical management, thus building patient and caregiver trust in their clinical decision-making^23 26 28 29 32 41 42^; larger-scale benefits, such as better antibiotic stewardship may follow.^23^ Fourth, pulse oximetry should not be rolled out in isolation if its full potential to improve the care of acutely febrile patients in LMICs is to be realised. Rather, it must be part of a systems approach incorporating reliable access to supplemental oxygen as well as strengthening of health infrastructure, sustainable provision of context-appropriate equipment and staff training, and continuous and comprehensive quality improvement. As such, pulse oximetry should not be seen as a simple but rather a complex intervention, with appropriate planning required to ensure all relevant components of the health system interact smoothly. Such efforts should be based on first principles of implementation science and findings from the wider evidence base, drawing on experiences of rolling out other complex interventions.^43 44^

Our review has identified several key issues which future research on this topic must address to increase confidence in the above conclusions and make them more generalisable. The first is the determination of age-appropriate and altitude-appropriate SpO_2_ thresholds below which escalation of care should be considered. This is particularly important in neonates and very young infants in whom moderate hypoxaemia has been shown to be associated with increased mortality,^5^ but for whom no recommendation exists in the IMCI guidance widely used in LMICs.^35^ Given the already low bar for escalation to higher level care in this patient group based on clinical assessment alone, lowering the oxygen saturation threshold for referral to <93% may not result in over-referral as much as it would for older children but the value added by pulse oximetry may also be reduced.^29^ This is important from a cost-effectiveness viewpoint, as optimal peripheral oxygen saturation measurement in neonates and very young infants requires extra investment in the form of neonatal probes. A similar lacuna also exists for patients who reside at high altitude, as their usual peripheral oxygen saturations may fall below the widely accepted cut-off for severe hypoxaemia of 90%.^45 46^ Related to these issues is the need to establish the accuracy of contemporary pulse oximeters in darker-skinned patients across a range of ages, disease states and oxygen saturations, given the conflicting findings in the literature on this issue.^47–49^ The second is the development of algorithmic diagnostic and prognostic tools incorporating pulse oximetry in conjunction with other clinically significant variables, tailored to the contexts and settings in which they will be used. This is essential to ensure safe clinical decision-making when these algorithms are used by health workers with limited skills, as well as to strike the necessary balance between early identification of the severely ill and maintaining the viability of often fragile secondary and higher-level care systems by avoiding over-referral.^14 50^ Third, there is a pressing need for well-designed health systems and implementation research evaluating pulse oximetry as part of a suite of quality-assured system-wide or facility-wide interventions. Such efforts should attempt to bridge the gap between the diagnostic and prognostic roles of pulse oximetry in a contextualised manner by basing outcome measures on proposed use-cases and building on previous work on strategies to improve its institutional adoption.^50 51^ This research should include adults, assess cost-effectiveness with robust economic evaluation methods, and include Asia, as high-quality evidence for the use of pulse oximetry pertinent to this region is sorely lacking. Fourth, less common roles for pulse oximetry, such as its use as a trust-building and communication aid between healthcare workers and patients or caregivers with limited health literacy should be further explored.^41^

While this review is, to our knowledge, the first to appraise pulse oximetry in the assessment of patients with acute febrile illness residing in resource-limited settings, it has several weaknesses. The greatest of these limitations is the heterogeneity in study design, interventions and endpoints, which renders robust statistical comparison of effect sizes in the already small number of studies impossible.^20^ Second, study quality was generally poor, and the populations and syndromes to which the findings are applicable limited. Third, while we have endeavoured to construct a comprehensive search strategy, such as making the primary focus of the review acute febrile illness rather than specific infective syndromes, snowballing and reaching out to major global health practitioners for grey literature, it is possible that not all relevant studies were included. However, a systematic review of articles published up to 2015 examining the effect of pulse oximetry on paediatric mortality, morbidity, length of stay and treatment and management changes in both HICs and LMICs identified only one study included in our review.^16^ This fact, in addition to the larger number of databases we searched, indicates a low probability of our search strategy missing important studies. Furthermore, framing this review around acute fever has the advantage of identifying studies applicable to healthcare workers in LMICs who may have limited diagnostic skills but who are expected to manage patients presenting with this non-specific symptom. Finally, while systematic reviews like ours are useful in synthesising evidence from sources with diverse methodologies and contexts, critical features and procedural details of the intervention of interest are not able to be described at length. Along with the need to consider the broader evidence base, as mentioned earlier, this limitation necessitates approaches such as Intervention Component Analysis or similar frameworks when translating the evidence presented here into practice.^52^

## Conclusions

When used for the assessment of acute febrile illness in LMICs, pulse oximetry may assist clinicians in diagnosing and treating severe paediatric respiratory illnesses and making referrals to higher-level care. It may also reduce mortality from this condition if used as part of a holistic package of interventions which takes local context into account. The evidence on which these conclusions are based is not widely generalisable and is of poor quality. Thus, health facilities or systems intending to implement pulse oximetry should also consider evidence from the wider implementation science literature, given the limitations of systematic reviews in synthesising evidence on systems approaches to specific complex interventions.

## Data Availability

Data are available upon reasonable request. All data relevant to the study are included in the article or uploaded as supplementary information.
