# Supplementary material for: The impact of pulse oximetry on diagnosis, management and outcomes of acute febrile illness in low-income and middle-income countries: a systematic review
Source: BMJ Glob Health. 2021 Nov 25;6(11):e007282. doi: 10.1136/bmjgh-2021-007282 (PMC8627405; doi:10.1136/bmjgh-2021-007282)
Supplement: Supplementary data [file bmjgh-2021-007282supp002.pdf]

SUPPLEMENTAL APPENDIX S2

Electronic database search strings

**DARE**
Pulse oximet* [All fields]

**PubMed**
"Oximetry"[MeSH] OR "pulse oximet*"[Title/Abstract]<br><br>AND<br><br>"fever"[MeSH] OR fever[Title/Abstract] OR febrile[Title/Abstract] OR pyrexia[Title/Abstract] OR "communicable diseases"[MeSH] OR "sepsis"[MeSH] OR "systemic inflammatory response syndrome"[MeSH] OR "pneumonia"[MeSH] OR "systemic inflammatory response syndrome"[Title/Abstract] OR SIRS[Title/Abstract] OR septic[Title/Abstract] OR infect*[Title/Abstract] OR respiratory[Title/Abstract] OR pneumonia[Title/Abstract]<br><br>AND<br><br>"Developing Countries"[MeSH] OR "Asia"[MeSH] OR "Africa"[MeSH] OR "Latin America"[MeSH] OR "South America" [MeSH] OR Asia[Title/Abstract] OR Africa[Title/Abstract] OR "South America"[Title/Abstract] OR "Latin America"[Title/Abstract] OR "Oceania"[Title/Abstract] OR "Australasia"[Title/Abstract] OR "Pacific"[Title/Abstract] OR "low income"[Title/Abstract] OR "middle income"[Title/Abstract] OR "low and middle income" OR "developing countr*"[Title/Abstract] OR "low resource"[Title/Abstract] OR "resource limited"[Title/Abstract]

**EMBASE**
Exp pulse oximetry/ or "pulse oximet*".mp.<br><br>AND<br><br>fever.mp. or exp fever/ or pyrexia.mp. or sepsis.mp. or exp sepsis/ or pneumonia.mp. or exp pneumonia/ or respiratory.mp. or exp systemic inflammatory response syndrome/ or exp infection/ or infection.mp.<br><br>AND<br><br>Exp middle income country/ or exp developing country/ or lmic.mp. or asia.mp. or exp Asia/ or exp Africa/ or africa.mp. or latin america.mp. or exp "South and Central America"/ or oceania.mp. or exp Pacific islands/ or australasia.mp.

**Web of Science (all databases)**
TS=("pulse oximet*")<br><br>AND<br><br>TS=(fever OR pyrexia OR sepsis OR SIRS OR febrile OR pneumonia OR infectio*) OR<br>TI=(sepsis OR septic OR fever OR febrile OR pyrexia<br>OR pneumonia OR infectio* OR SIRS OR systemic inflammatory response syndrome OR respiratory)<br>OR<br>AB=(sepsis OR septic OR fever OR febrile OR pyrexia<br>OR pneumonia OR infectio* OR SIRS OR systemic inflammatory response syndrome OR respiratory)<br><br>AND<br><br>TS=(developing countr* OR low income OR middle income) OR<br>TI=(developing countr* OR low income OR middle income OR Asia OR Africa OR South America OR Lat in America OR Oceania OR Australasia OR Pacific OR LMIC OR resource limited OR low resource) OR<br>AB=(developing countr* OR low income OR middle income OR Asia OR Africa OR South America OR La tin America OR Oceania OR Australasia OR Pacific OR LMIC OR resource limited OR low resource)

**CINAHL**
MM pulse oximetry OR MM pulse oximeter OR AB pulse oximet*<br><br>AND<br><br>MM sepsis OR MM fever OR MM pyrexia OR MM pneumonia OR MM systemic inflammatory response syndrome OR MM infectious disease OR MM infection OR AB (sepsis or septic) OR AB infect* OR AB pneumonia OR AB (fever or febrile or pyrexia) OR AB (sirs or systemic inflammatory response syndrome) OR AB respiratory<br><br>AND<br><br>MM lmic or low income countries or middle income countries or developing countries OR<br>AB asia OR AB africa OR AB (south america or latin america or central america) OR AB pacific OR AB oceania OR AB australasia OR AB low resource

**Global Health**
pulse oximetry.mp.<br><br>AND<br><br>(sepsis or septic or fever or pyrexia or febrile or pneumonia or infectio* or SIRS or systemic inflammatory response syndrome or respiratory).mp.

**Global Index Medicus**
(mh:(oximetry)) OR (tw:("pulse oximet*"))<br><br>AND<br><br>(mh:(fever)) OR (mh:(sepsis)) OR (mh:(pneumonia)) OR (mh:("communicable diseases")) OR (mh:("systemic inflammatory response syndrome")) OR (tw:(fever)) OR (tw:(pyrexia)) OR (tw:(febrile)) OR (tw:("systemic inflammatory response syndrome")) OR (tw:(SIRS)) OR (tw:(septic)) OR (tw:(infect*)) OR (tw:(respiratory)) OR (tw:(pneumonia))

**CENTRAL**
#1: MeSH descriptor: [oximetry] explode all trees<br><br>#2: MeSH descriptor: [fever] explode all trees<br>#3: MeSH descriptor: [pyrexia] explode all trees<br>#4: MeSH descriptor: [sepsis] explode all trees<br>#5: MeSH descriptor: [pneumonia] explode all trees<br>#6: MeSH descriptor: [communicable diseases] explode all trees<br>#7: MeSH descriptor: [systemic inflammatory response syndrome] explode all trees<br>#8: (fever OR febrile OR pyrexia OR “systemic inflammatory response syndrome” OR SIRS OR septic OR infect* OR respiratory OR pneumonia):ti,ab,kw<br><br>#9: #2 OR #3 OR #4 OR #5 OR #6 OR #7 OR #8<br><br>#10: #1 AND #9

All databases were last accessed on 29 June 2021.
