# Supplementary material for: The impact of pulse oximetry on diagnosis, management and outcomes of acute febrile illness in low-income and middle-income countries: a systematic review
Source: BMJ Glob Health. 2021 Nov 25;6(11):e007282. doi: 10.1136/bmjgh-2021-007282 (PMC8627405; doi:10.1136/bmjgh-2021-007282)
Supplement: Supplementary data [file bmjgh-2021-007282supp003.pdf]

SUPPLEMENTAL APPENDIX S3

Technical Advisory Panel members

Name	Position	Email
Mike English	Professor of International Child Health, University of Oxford	menglish@kemri-wellcome.org
Jennifer Daily	Independent consultant in *in vitro* diagnostics and global health markets; formerly Manager – Diagnostics Access Initiatives, CHAI (Clinton Health Access Initiative)	jenniferanndaily@gmail.com
Arjen Dondorp	Professor of Tropical Medicine, University of Oxford	arjen@tropmedres.ac
David Bell	Independent consultant in global health, diagnostics, and infectious disease; formerly Head of Program – Malaria and Acute Febrile Syndrome, FIND (Foundation for Innovative New Diagnostics)	bell00david@gmail.com

Authors and practitioners proposed by Technical Advisory Panel

Authors – for publication list searches	Practitioners – for grey literature recommendations
Amy Sarah Ginsburg	Yasir bin Nisar
Karin Källander	Martin Willi Weber
Kevin Baker	Mike Ruffo
Quique Bassat	Cindy McWhorter
Mark Ansermino	Ann Detjen
	Iain Wilson
	Smita Kumar
	Rasa Izadnegahdar
	Felix Lam
