# Supplementary material for: The impact of pulse oximetry on diagnosis, management and outcomes of acute febrile illness in low-income and middle-income countries: a systematic review
Source: BMJ Glob Health. 2021 Nov 25;6(11):e007282. doi: 10.1136/bmjgh-2021-007282 (PMC8627405; doi:10.1136/bmjgh-2021-007282)
Supplement: Supplementary data [file bmjgh-2021-007282supp004.pdf]

SUPPLEMENTAL APPENDIX S4

Studies rejected at full-text review stage

No.	Study reference	Reason for rejection
1	Tanen DA, Trocinski DR. (2002) The use of pulse oximetry to exclude pneumonia in children. *Am J Emerg Med* 6:521-3.	High-income country setting
2	Starr N, Rebollo D, Asemu YM, et al. (2020) Pulse oximetry in low-resource settings during the COVID-19 pandemic. *Lancet Glob Health* 8:e1121-2.	Peri-operative setting
3	Enoch AJ, English M, Clinical Information Network, McGivern G, Shepperd S. (2019) Variability in the use of pulse oximeters with children in Kenyan hospitals: A mixed-methods analysis. PLoS Med 16:e1002987.	Participants not in target population
4	Cummings MJ, Goldberg E, Mwaka S, et al. (2017) A complex intervention to improve implementation of World Health Organization guidelines for diagnosis of severe illness in low-income settings: a quasi-experimental study from Uganda. *Implement Sci* 12:126.	Participants not in target population
5	Tolla HS, Asemere YA, Belete AB, et al. (2018) Use of pulse oximetry for diagnosis of hypoxemia and monitoring of children with pneumonia: A descriptive study from Ethiopia. *Am J Trop Med Hyg* 99:409.	No valid comparator group
6	Floyd J, Wu L, Burgess DH, Izadnegahdar R, Mukanga D, Ghani AC. (2015) Evaluating the impact of pulse oximetry on childhood pneumonia mortality in resource-poor settings. *Nature* 528:S53-9.	Modelling study
7	Rees CA, Basnet S, Gentile A, et al. (2020) An analysis of clinical predictive values for radiographic pneumonia in children. *BMJ Global Health* 5:e002708.	Modelling study
8	McCollum ED, King C, Colbourn T, et al. (2019) Pulse oximetry in paediatric primary care in low-income and middle-income countries. *Lancet Respir Med* 7:1001-2.	Opinion
9	Matata L, Baker K, Mucunguzi A, Posada M, Habte T, Kallander K (2015) Acceptability of selected respiratory rate counters and pulse oximeters for use by frontline health workers in the detection of pneumonia symptoms in children in sub-Saharan Africa and Southeast Asia. *Am J Trop Med Hyg* 93(4 Suppl):543.	Abstract with no results
10	Baker K, Mucunguzi A, Matata L, Posada M, Habte T, Kallander K (2015) Accuracy of selected devices for the detection of pneumonia symptoms – results from laboratory testing and field evaluations of respiratory rate counters and pulse oximeters with frontline health workers in sub-Saharan Africa and Southeast Asia. *Am J Trop Med Hyg* 93(4 Suppl):543.	Abstract with no results

11	Hamid MA, Chandna A, Siddiqui S, Fayyaz J (2016) Pulse oximetry: A reliable and cost effective screening tool in children with pneumonia for developing countries. *J Pak Med Assoc* 66:1015-8.	Narrative review
12	Herbert LJ, Wilson IH (2012) Pulse oximetry in low-resource settings. *Breathe* 9:90-7.	Narrative review
13	Duke T, Subhi R, Peel D, Frey B (2009) Pulse oximetry: Technnology to reduce child mortality in developing countries. *Ann Trop Paediatr* 29:165-75.	Narrative review
