# Supplementary material for: The impact of pulse oximetry on diagnosis, management and outcomes of acute febrile illness in low-income and middle-income countries: a systematic review
Source: BMJ Glob Health. 2021 Nov 25;6(11):e007282. doi: 10.1136/bmjgh-2021-007282 (PMC8627405; doi:10.1136/bmjgh-2021-007282)
Supplement: Supplementary data [file bmjgh-2021-007282supp005.pdf]

GENERAL INFORMATION

Extraction date
Citation
Publication type

ELIGIBILITY

Eligibility criteria met
Type of study
Participants
Intervention
Comparison
Outcome measures

METHODS

Aim
Design
Unit of allocation
Start date
End date
Duration of participation
Ethical approval needed/obtained

PARTICIPANTS

Population
Setting
Inclusion criteria
Exclusion criteria
Method of recruitment
Informed consent
Total population at start
Clusters
Baseline imbalances
Withdrawals and exclusions
Demographics

GROUPS

Intervention and comparison
Description of intervention
Treatment duration
Timing
Delivery
Providers
Co-intervention
Economic information
Resource requirements
Integrity of delivery
Compliance

RELEVANT OUTCOMES
Outcome name
Time points measured
Time points reported
Outcome definition
Person measuring/reporting
Outcome validated
Imputation of missing data
Assumed risk estimate
Power

OTHER
Funding sources
Conflicts of interest

DATA AND ANALYSIS
Results
Any other results
Missing participants
Unit of analysis
Statistical methods and appropropriateness
Reanalysis required
Reanalysis possible

CONCLUSIONS
Key findings
Correspondence required
